# Three Novel *De Novo ZEB2* Variants Identified in Three Unrelated Chinese Patients With Mowat-Wilson Syndrome and A Systematic Review

**DOI:** 10.3389/fgene.2022.853183

**Published:** 2022-05-12

**Authors:** Youqing Fu, Wanfang Xu, Qingming Wang, Yangyang Lin, Peiqing He, Yanhui Liu, Haiming Yuan

**Affiliations:** ^1^ Affiliated Dongguan Maternal and Child Health Care Hospital, Southern Medical University, Dongguan, China; ^2^ Dongguan Institute of Reproductive and Genetic Research, Dongguan, China

**Keywords:** Mowat-Wilson syndrome, ZEB2, epilepsy, hirschsprung disease, happy demeanor

## Abstract

**Background:** *ZEB2* gene mutations or deletions cause Mowat-Wilson syndrome (MWS), which is characterized by distinctive facial features, global developmental delay, intellectual disability, epilepsy, friendly and happy personalities, congenital heart disease, Hirschsprung disease and multiple congenital anomalies. Currently, more than 300 MWS patients have been described in the literature, and nearly 280 variants in *ZEB2* have been identified.

**Methods:** In this study, we report three unrelated Chinese patients presenting multiple congenital anomalies that were consistent with those of MWS. Whole-exome sequencing (WES) was used to identify the causative variants.

**Results:** WES identified two novel *de novo* frameshift variants in *ZEB2* (NM_014795.4:c.2136delC, p. Lys713Serfs*3 and c.2740delG, p. Gln914Argfs*16) in patients 1 and 2, respectively, and a novel *de novo* splicing variant in *ZEB2* (NM_014795.4:c.808-2delA) in patient 3, all of which were confirmed by Sanger sequencing. Next, we systematically reviewed the clinical characteristics of Chinese and Caucasian MWS patients. We revealed a higher incidence of constipation in Chinese MWS patients compared to that previously reported in Caucasian cohorts, while the incidence of Hirschsprung disease and happy demeanor was lower in Chinese MWS patients and that epilepsy in Chinese MWS patients could be well-controlled compared to that in Caucasian MWS individuals.

**Conclusion:** Our study expanded the mutation spectrum of *ZEB2* and enriched our understanding of the clinical characteristics of MWS. Definitive genetic diagnosis is beneficial for the genetic counseling and clinical management of individuals with MWS.

## Introduction

Mowat-Wilson syndrome (MWS; OMIM #235730) is a rare autosomal dominant genetic disorder, characterized by distinctive facial features, global developmental delay, intellectual disability, epilepsy, congenital heart disease, Hirschsprung disease, corpus callosum agenesis, short stature, genitourinary anomalies, hypotonia and friendly and happy personalities (Mowat et al., 1998; Wakamatsu et al., 2001; Mowat et al., 2003; Wilson et al., 2003; Zweier et al., 2006; Garavelli and Mainardi, 2007; Evans et al., 2012; Bourchany et al., 2015). MWS is caused by pathogenic variants or the deletion of *ZEB2* (OMIM# 605802) at 2q22.3 (Cacheux et al., 2001). *ZEB2* encodes the zinc finger E-box binding homeobox 2 protein, which consists of 1,214 amino acids. The ZEB2 protein is a member of the family of δEF1/Zfh-1, and it contains a SMAD-binding domain, a homeodomain-like sequence, and two separate clusters of zinc fingers at the N-terminus and the C-terminus (Remacle et al., 1999). The ZEB2 protein interacts with SMAD proteins and functions as a transcriptional repressor in response to TGF-β signaling (Verschueren et al., 1999). SMAD proteins are cytoplasmic mediators that are tightly controlled and play an important role in transmitting TGF-ß signals from cell surface receptors to the nucleus (Verschueren et al., 1999). ZEB2 is expressed in most human tissues and is essential for the development and migration of neural crest cells (Van de Putte et al., 2003), heart separation and midline development during early embryogenesis (Vandewalle et al., 2009).

To date, more than 300 individuals with MWS have been reported in the literature, and approximately 280 variants in *ZEB2* have been identified (HGMD database; Wei et al., 2021; Zhang et al., 2021; Hu et al., 2020; Ma et al., 2020; Zou et al., 2020). However, Chinese MWS individuals are relatively less described, and only 27 MWS cases and 23 pathogenic *ZEB2* variants have been reported for Chinese individuals (Balasubramaniam et al., 2010; Jiang et al., 2016; Hu et al., 2018; Wang et al., 2019; Ho et al., 2020; Hu et al., 2020; Ma et al., 2020; Wu et al., 2020; Zou et al., 2020; Wei et al., 2021; Zhang et al., 2021). Furthermore, phenotypic differences between Chinese and Caucasian MWS individuals have been less delineated. Recently, Ho et al. (2020) summarized for the first time the clinical features and molecular findings of a small Chinese MWS cohort (15 patients) and compared them to those previously reported in Caucasian cohorts. Here, we report three novel *ZEB2* variants in three unrelated Chinese MWS patients and systematically review the clinical characteristics of Chinese and Caucasian MWS individuals.

## Materials and Methods

### Ethical Compliance

This study was approved by the Ethics Committee of Dongguan Maternal and Child Health Care Hospital. Written informed consent was obtained from the legal guardian for the publication of any potentially identifiable images or data included in this article.

### Whole-Exome Sequencing

Whole-exome sequencing (WES) of the patients was performed to screen for causal variants. Sequencing was performed on the NextSeq500 platform (Illumina) according to the manufacturer’s protocols. Clinic Sequence Analyzer (CSA) software was used for biological analysis and interpretation. The pathogenicity of the sequence variants was evaluated in accordance with the American College of Medical Genetics and Genomics/Association for Molecular Pathology (ACMG/AMP) guidelines (Richards et al., 2015).

## Results

### Patient One

**FIGURE 1:**
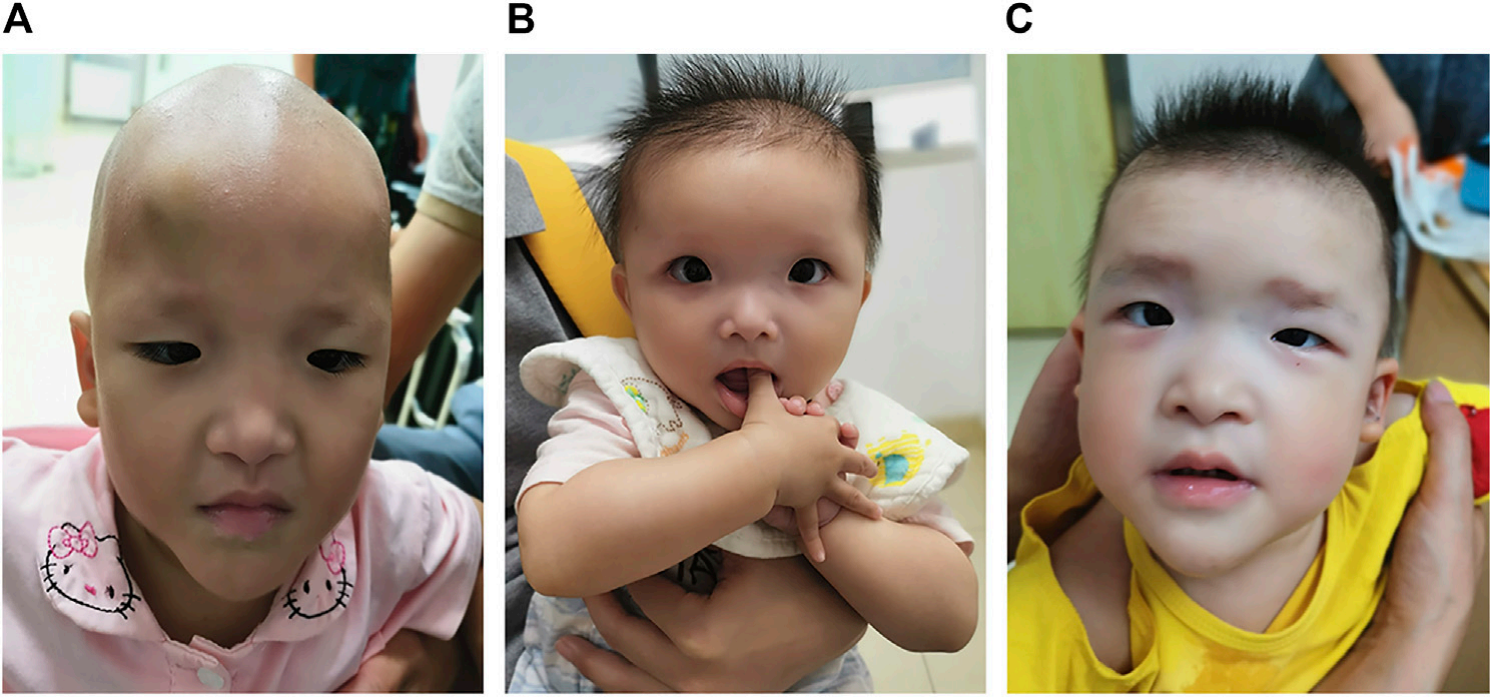
Photographs of patients with Mowat–Wilson syndrome. Note microcephaly, sparse hair and eyebrows, deep-set large and widely spaced eyes, low-set and upturned earlobes, a saddle nose with a rounded nasal tip and a pointed chin in patient 1 **(A)**. Note microcephaly, frontal bossing, square-shaped high forehead, sparse hair and eyebrows, deep-set large and widely spaced eyes, auricle dysplasia, a saddle nose with a rounded nasal tip, open mouth expression and a pointed chin in patient 2 **(B)**. Note microcephaly, frontal bossing, a square-shaped high forehead, sparse hair, flaring eyebrows, hypertelorism, auricle dysplasia, low-set and upturned earlobes, a saddle nose with a rounded nasal tip, M-shaped upper lip, open mouth expression and a pointed chin in patient 3 **(C)**.

The patient was the second child of a healthy nonconsanguineous couple and her older sister was healthy. She was born by vaginal delivery at 38 weeks of gestation. Birth length was 50 cm and birth weight was 3.0 kg. She had hypotonia. At 1 month of age, she had a diagnosis of Hirschsprung disease with surgical removal of the aganglionic segment. At the age of 1 year and 8 months, she first presented with epilepsy, which was triggered by fever. It lasted for 10 min with spontaneous remission. Two months later, she again suffered from epilepsy, which lasted for approximately 3 min with spontaneous remission. The patient was 1 year and 10 months old at the time of molecular evaluation. Her height was 80 cm (<−1 SD), weight was 8.8 kg (<−2 SD) and head circumference was 42 cm (<−3 SD). She had distinctive facial features including microcephaly, sparse hair and eyebrows, deep-set large and widely spaced eyes, low-set and upturned earlobes, a saddle nose with a rounded nasal tip and a pointed chin (Figure 1A).

On recent physical examination at the age of 4 years, she still displayed persistent growth delay. Her height was 95 cm (<−2 SD), her weight was 12.5 kg (<−2 SD), and her head circumference was 45 cm (<−3 SD). The development milestones were delayed. She raised her head at 5 months, sat alone at 1 year and walked without assistance at 2 years and 6 months. Her cognitive competence was significantly lower than her peers, with an intelligence quotient of 50 by the Wechsler Preschool and Primary Scale of Intelligence (WPPSI-III). She had no language development yet. She always displayed a happy affect and sociable demeanor as well as timid behavior. She had chronic constipation and indigestion.

**FIGURE 2:**
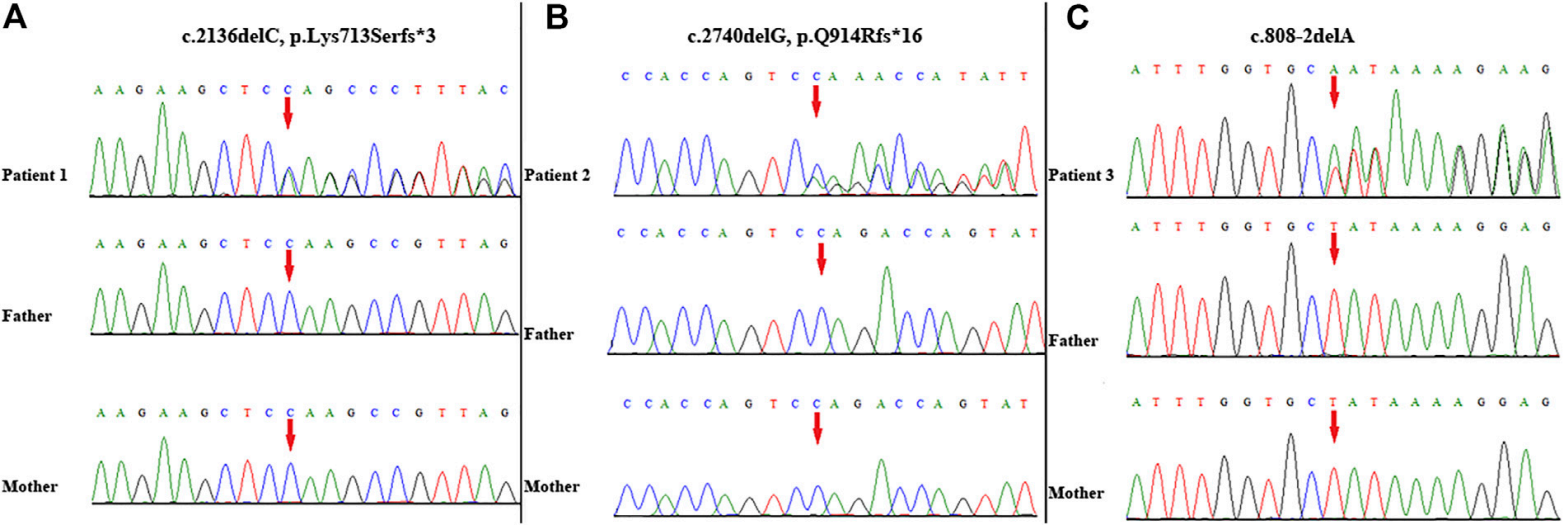
Variant identification by Sanger sequencing. A *de novo* frameshift variant c. 2136delC, p. Lys713Serfs*3 in *ZEB2* was detected in patient 1 **(A)**; A *de novo* frameshift variant c. 2740delG, p. Q914Rfs*16 in *ZEB2* was detected in patient 2 **(B)**; A *de novo* splicing variant c. 808-2delA in *ZEB2* was detected in patient 3 **(C)**. The red arrow indicates the variant site.

A novel heterozygous frameshift variant (NM_014795.4:c.2136delC, p. Lys713Serfster3) in *ZEB2* was identified in this patient. This variant was not detected in her parents; thus, it was a *de novo* event (Figure 2A). It was not present in either the Genome Aggregation Database or 1,000 Genomes Project database. Thus, this variant was categorized as clinically pathogenic according to ACMG/AMP guidelines (PVS1 + PS2 + PM2) (PVS: pathogenic very strong; PS: pathogenic strong; PM: pathogenic moderate) (Richards et al., 2015).

### Patient Two

The proband was the first child of healthy unrelated parents. She was born by cesarean section at 39 weeks of gestation. Her birth length was 50 cm and birth weight was 3.2 kg. She had hypotonia and feeding difficulties. At 5 months of age, she had a diagnosis of Hirschsprung disease with surgical removal of the intestine lacking ganglion cells and of the proximal colon with obvious hypertrophy. Her height was 68.9 cm (<−2 SD), weight was 7.1 kg (<−2 SD) and head circumference was 41 cm (<−2 SD) at the age of 10 months. She had distinctive facial features including microcephaly, frontal bossing, square-shaped high forehead, sparse hair and eyebrows, deep-set large and widely spaced eyes, auricle dysplasia, a saddle nose with a rounded nasal tip, open mouth expression and a pointed chin (Figure 1B). She always displayed a happy demeanor and enjoyed smiling and clapping hands. Brain magnetic resonance imaging (MRI) showed corpus callosum hypoplasia. She could not sit alone and had no language development at the age of 1 year. She had chronic constipation.

WES identified a novel frameshift variant (NM_014795.4:c.2740delG, p. Gln914Argfs*16) in *ZEB2* in this patient. This variant was not present in either the Genome Aggregation Database or 1,000 Genomes Project. Subsequent targeted Sanger sequencing confirmed the *de novo* origin of the variant (Figure 2B). Thus, this variant can be categorized as pathogenic according to ACMG/AMP guidelines (PVS1 + PS2 + PM2) (PVS: pathogenic very strong; PS: pathogenic strong; PM: pathogenic moderate) (Richards et al., 2015).

### Patient Three

The proband was the second child of healthy unrelated parents and her 8-year-old sister was healthy. The patient was born by vaginal delivery at 38 weeks of gestation. Her birth length was 49 cm and birth weight was 3 kg. She had hypotonia and feeding difficulties. A surgical operation was carried out for the patient due to Hirschsprung disease at 4 months old. At the age of 3 years and 10 months, she was referred to our clinic due to delayed development milestones. She sat alone at 2 years, could not walk independently and had no language development until now. Her height was 86 cm (<−2 SD), her weight was 11 kg (<−2 SD) and her head circumference was 45 cm (<−2 SD). She had distinctive facial features, including microcephaly, frontal bossing, a square-shaped high forehead, sparse hair, flaring eyebrows, hypertelorism, auricle dysplasia, low-set and upturned earlobes, a saddle nose with a rounded nasal tip, M-shaped upper lip, open mouth expression and a pointed chin (Figure 1C). She demonstrated poor eye contact, had no response to simple instructions, displayed social difficulties and stereotyped behaviors as well as restricted interests, such as slapping tables, biting toys and eating fingers. She met the clinical diagnostic criteria for autism based on the Autism Behavior Checklist, Childhood Autism Rating Scale, and Modified Checklist for Autism in Toddlers Revised. She had chronic constipation. MRI showed corpus callosum hypoplasia.

A novel heterozygous splicing variant (NM_014795.4:c.808-2delA) in intron 6 of *ZEB2* was identified in this patient. Subsequent targeted Sanger sequencing confirmed the *de novo* origin of the variant (Figure 2C). It was not present in either the Genome Aggregation Database or 1,000 Genomes Project database. Thus, this variant was categorized as clinically pathogenic according to ACMG/AMP guidelines (PVS1 + PS2 + PM2) (PVS: pathogenic very strong; PS: pathogenic strong; PM: pathogenic moderate) (Richards et al., 2015).

## Discussion

To date, more than 300 individuals with MWS have been reported in different regions of the world. And nearly 280 variants in *ZEB2* have been identified (HGMD database; Wei et al., 2021; Zhang et al., 2021; Hu et al., 2020; Ma et al., 2020; Zou et al., 2020) (Supplementary Table S1). However, Chinese MWS individuals have been infrequently reported (Supplementary Table S2). Phenotypic differences between Chinese and Caucasian MWS patients have been described even less frequently. In this study, we identified three novel *de novo ZEB2* variants (p.Lys713Serfs*3, p. Gln914Argfs*16, c.808-2delA) in three unrelated Chinese patients. All patients displayed peculiar facial features, global developmental delay, intellectual disability, microcephaly, short stature, Hirschsprung disease and other anomalies that were consistent with those of MWS. The newly identified variants expand the *ZEB2* mutation spectrum and could improve the molecular diagnosis of MWS.

**FIGURE 3:**
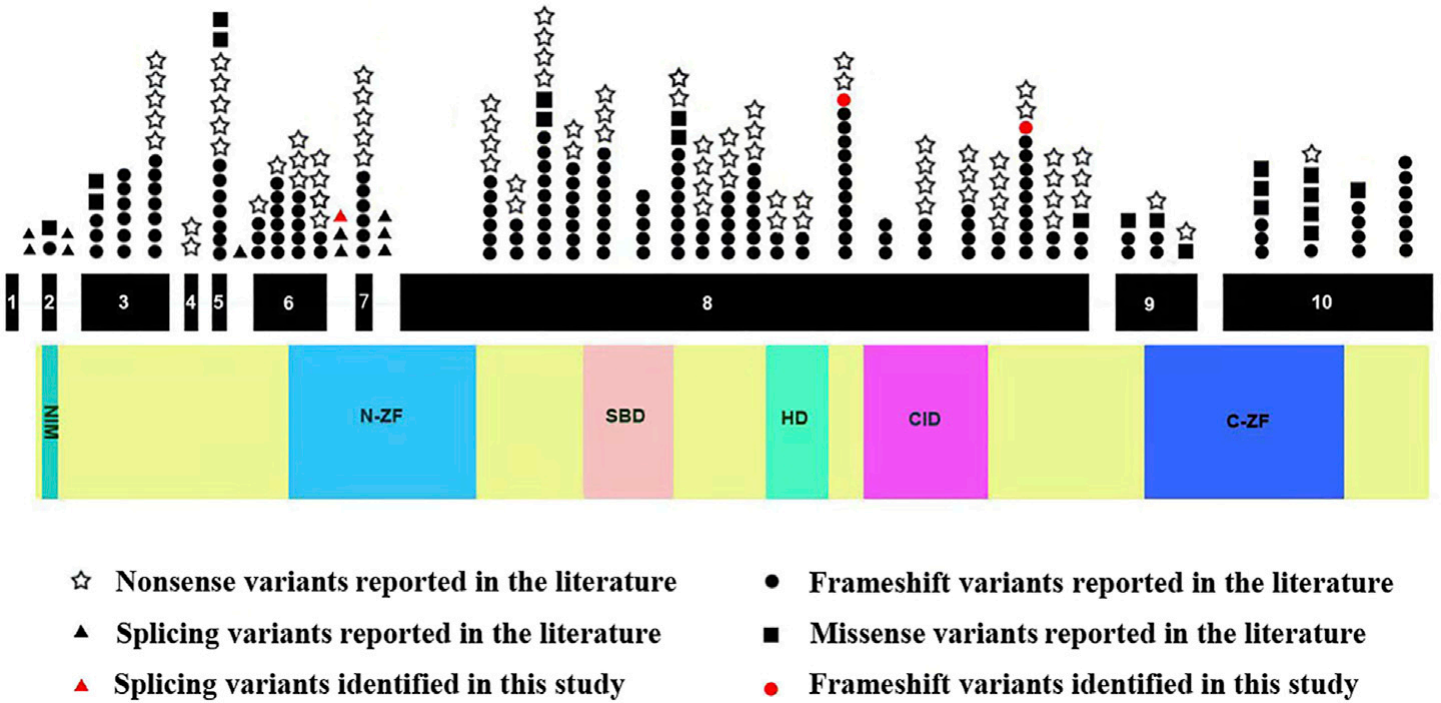
Schematic representation of *ZEB2* variants identified to date. The structure of *ZEB2* contained 10 exons (black rectangles), introns (green horizontal line); lower side: the ZEB2 protein domains: NIM: NuRD-Interacting Motif (14–22); N-ZF: N-terminal Zinc Finger clusters (213–304); SBD: Smad-Binding Domain (437–487); HD: Homeodomain-like Domain (651–700); CID: CtBP-Interacting Domain (757–863); C-ZF: C-terminal Zinc Finger clusters (1,001–1,076).

Next, we systematically reviewed all *ZEB2* variants and clinical phenotypes in Chinese and Caucasian MWS patients. A total of 282 variants, including the 3 novel variants identified in our study, were identified. *ZEB2* frameshift, nonsense, splicing and missense variants causing MWS account for 59.6, 29.1, 3.9 and 7.4%, respectively. Mutation hotspots were identified in exons 8 (56.4%), 6 (8.9%), 10 (8.5%) and 3 (8.2%). These variants were distributed to the N-ZF domain (11.3%), C-ZF domain (6.4%), CID domain (6.0%), SBD domain (5.7%), HD domain (2.8%) and NIM domain (0.7%). We found that all splicing variants were located in the N-ZF domain and the missense variants were mainly located in the C-ZF domain (Figure 3).

We analyzed the phenotypic differences between Chinese and Caucasian MWS patients. The incidence of each phenotype in Chinese and Caucasian MWS patients is summarized in Table 1. Epilepsy is a main feature of MWS. Up to 25% of affected Caucasian MWS individuals have epilepsy that is difficult to control or refractory to conventional anti-seizure medications (Ivanovski et al., 2018). However, only 19% of Chinese MWS patients have epilepsy that reaches a nearly epilepsy-free state by antiepileptic drugs, and the remaining patients with epilepsy have spontaneous remission without treatment. Hirschsprung disease is known to be more prevalent among Asian populations in general, with an incidence of 2.8 in 10,000 (Torfs, 1998). Based on the genetic background, the incidence of Hirschsprung disease in Chinese MWS patients (37.9%) should be higher than that in Caucasian MWS patients (44.2%). Here, all three patients in our study had anatomically proven Hirschsprung disease at an early age. Therefore, the possibility should be considered that some MWS patients who were initially diagnosed with Hirschsprung disease at an early age do not undergo molecular diagnosis due to the clinicians’ insufficient recognition of this disorder, as discussed below. We also observed that the incidence of constipation in Chinese MWS patients (51.7%) was markedly higher than that in Caucasian MWS patients (29%). The possibility was not excluded that some Chinese MWS patients with constipation who may in fact suffer from Hirschsprung disease did not receive the appropriate diagnosis since biopsies was not performed. However, it was difficult to compare the difference between the numbers of biopsies effected for Caucasian and Chinese MWS patients. Friendly and happy personalities were also a prominent feature of MWS. The incidence of this feature in Caucasian MWS patients (47%) was significantly higher than that in Chinese MWS patients (23.3%). Two of three patients in our study displayed this phenotype. Thus, further studies are required to determine whether there is a genuine ethnicity-related effect in the MWS phenotypes or a statistical bias arising from insufficient samples.

**TABLE1:** Incidence of main clinical features in Chinese and Caucasian MWS individuals.

Features	Chinese	Caucasian@
Gender (male:female)	12:18	183:161
Distinctive facial features	30/30 (100%)	81/87 (93.1%)
Microcephaly	20/30 (66.7%)	244/314 (77.7%)
Seizure	22/30 (73.3%)	241/307 (78.5%)
Short stature	12/30 (40.0%)	70/151 (46.4%)
Hirschsprung disease	10/30 (33.3%)	148/335 (44.2%)
Constipation	14/30 (46.7%)	90/310 (29%)
Congenital heart disease	19/30 (63.3%)	193/332 (58.1%)
Friendly and happy personalities	7/30 (23.3%)	32/68 (47.0%)

@adapted from Ivanovski et al. (2018).

Autism has rarely been described in MWS individuals to date. However, it has been reported that some patients with MWS exhibit motor stereotypies, such as repeated movements of the hands and head. Other patients have been reported to be fascinated by turning the pages of books and magazines (Adam et al., 2006). In a previously published study, stereotyped behaviors were noted to be more frequent, with a higher score for the items “flicks taps twirls objects” (DBC Item 25) and “switches lights on and off or similar repetitive activity” (DBC Item 72) (Evans et al., 2012). Repetitive and stereotyped behaviors were also described in some MWS individuals (Bonanni et al., 2017). Recently, Ho et al. (2020) described the first case series for Chinese MWS patients, four of which demonstrated stereotypic hand movements. Wu et al. (2020) identified a novel *de novo ZEB2* variant (c.547dupC, p. L183fs) in a 5-years-old female patient who displayed autistic phenotypes, neurodevelopmental delay and other anomalies. Unfortunately, specialized autism assessments were not performed for these patients. In our study, patient three did not speak and had poor eye contact, social difficulties and stereotyped behaviors. She met the clinical diagnostic criteria for autism based on the Childhood Autism Rating Scale and Autism Behavior Checklist. Thus, our report identified a case of MWS with autistic behavior, described in terms of both the detailed clinical manifestations and specialized autism assessments. Whether autism is truly part of the spectrum of MWS or is a coincidental secondary diagnosis due to the frequency of autism spectrum disorders in the general population remains to be observed.

The incidence of MWS was estimated to be 1/50,000–1/70,000 live births (Ghoumid et al., 2013), whereas, the incidence of MWS in the Chinese population seemed to be lower than that in the Caucasian population since only 30 Chinese MWS individuals have been reported. The main reason for this was that formal clinical diagnostic criteria for MWS had not been established, and clinicians lack an understanding of this disorder, which could lead to a lack of molecular diagnoses in most cases. However, the facial features of MWS are recognizable and, when accompanied by other features of the disorder (e.g., Hirschsprung disease and/or chronic constipation, intellectual disability/developmental delay), can establish the clinical diagnosis. Thus, we suggest that patients with clinical features suggestive of MWS should be evaluated by experienced clinical specialists and geneticists to help improve the diagnosis of this disorder.

In conclusion, we identified three novel variants in *ZEB2* in three unrelated Chinese individuals with MWS, which expands the mutation spectrum of *ZEB2*. Next, we systematically reviewed the phenotypic characteristics of Chinese and Caucasian MWS individuals. These findings will contribute to enriching our understanding, clinical management and genetic counseling of MWS, which needs to be explored further.

## Data Availability

The datasets for this article are not publicly available due to concerns regarding participant/patient anonymity. Requests to access the datasets should be directed to the corresponding author.
